# Leucine mediates cognitive dysfunction in early life stress-induced mental disorders by activating autophagy

**DOI:** 10.3389/fncel.2022.1060712

**Published:** 2023-01-04

**Authors:** Xiaotian Wang, Xue Wang, Fang Xie, Zhaowei Sun, Bomin Guo, Feng Li, Shida Wang, Ying Wang, Yingrui Tian, Yun Zhao, Lingjia Qian

**Affiliations:** Laboratory of Stress Medicine, Beijing Institute of Basic Medical Sciences, Beijing, China

**Keywords:** maternal separation stress, cognitive impairment, leucine, cerebrospinal fluid, autophagy, mental disorders

## Abstract

**Objectives:**

To explore the relationship between leucine in cerebrospinal fluid (CSF) and cognitive dysfunction in rats with early life stress (ELS) induced mental illness, and pathophysiological mechanism involved.

**Methods:**

The maternal separation (MS), an animal paradigm used widely as a preclinical model of ELS which is one of the important risk factors for mental disorders. Behavioral experiments including open-field test, sucrose preference, object recognition and Morris water maze tests, Nissl staining, transmission electron microscopy and WES were employed in the present study.

**Results:**

The behavioral results showed that MS rats were more prone to cognitive impairment and depression-and-anxiety-like behaviors than controls, including spatial self-exploration ability, memory ability, and spatial learning and memory function. Nissl staining analysis indicated that the number of neurons in the CA1 and CA3 regions of the hippocampus significantly decreased and the arrangement of nerve cells was abnormal. The leucine levels were decreased in the CSF of MS rats and highly correlated with the number of hippocampal neurons, and yet leucine supplementation improved the degree of MS-induced cognitive impairment. Furthermore, there were autophagosomes in the hippocampus of the low-leucine diet rats of the control and MS group but not in the high-leucine diet MS group by transmission electron microscopy. The protein expression of Beclin-1 in the hippocampus was significantly increased in the MS normal diet group and MS low-leucine diet group, yet decreased in the MS high-leucine diet group compared with the MS low-leucine diet group. Meanwhile, the Bcl-2/Bax ratio was significantly decreased in the control low-leucine diet group, MS normal diet group and MS low-leucine diet group. Ultimately, *in vitro* experiments suggested that leucine deficiency could activate neuronal autophagy including enhanced LC3II/LC3I and mRFP-GFP-LC3, which was consistent with the *in vivo* results, and the cell apoptosis rate and lactate dehydrogenase (LDH) cytotoxicity were also increased with leucine deficiency, while the above effects could be partly reversed by autophagy inhibitor treatment.

**Conclusions:**

MS model caused adult male rats to be susceptible to cognitive dysfunction, which may regulate autophagy in hippocampal neurons through leucine metabolism in CSF.

## Introduction

The developing brain is highly sensitive to stressful stimuli early in life, which can lead to changes in the number, structure, and function of neurons in the brain that persist into adulthood (Schäble et al., 2007). Adverse experiences early in life have been recognized as an important public health issue, especially for children left behind and orphans in adulthood (Nishi, 2020). The maternal separation (MS) model is the most intensively studied model of early life stress in mammals (Vetulani, 2013), and studies have shown that repeated 3–24-h periods of MS early in life lead to cognitive and emotional impairment in adulthood (Aisa et al., 2007; Kosten et al., 2007).

As the main cognitive module, learning and memory are closely associated with the hippocampus (Das et al., 2019), including a single synaptic pathway that projects directly from the third neuron in the entorhinal cortex to area CA1 and three synaptic pathways that project from the second neuron in the entorhinal cortex to the dentate gyrus (DG) and then to area CA3 via mossy fibers, so the number and arrangement of neurons in each region of the hippocampus are closely related to memory (Antunes and Biala, 2012). Cerebro-spinal fluid (CSF) is an important internal environment for brain survival and function, reflecting real-time dynamic information on cellular metabolism and function in the brain (Crews et al., 2009; Rahimi and Woehrer, 2017). The study of metabolic biomarkers in CSF provides important insights into the diagnosis and treatment of depression, schizophrenia, Alzheimer’s disease, and other diseases (Liguori et al., 2022).

Maternal separation (MS) leads to disturbance of neuroendocrine homeostasis in the body, and structural and functional damage to brain nerve cells occurs, inducing cognitive impairment and emotional abnormalities (Pervanidou and Chrousos, 2018). Metabolic dysfunction is an important feature of stress injury, and changes in amino acids are particularly evident (Cui et al., 2020). However, the precise pharmacological targets and effective therapeutic approaches according to amino acids metabolism feature after MS are still not fully understood so far. Recent studies have found that the severity of depression is significantly associated with elevated levels of proline in the blood of animals and that targeted regulation of dietary proline levels can effectively improve depressive states (Mayneris-Perxachs et al., 2022).

Leucine is a branched-chain amino acid (BCAA) that participates in the “glutamate-branch amino acid cycle shuttling” between neurons and astrocytes, avoiding potentially toxic accumulation of high glutamate concentrations through the metabolism of intermediate branched-chain keto acids (Murín and Hamprecht, 2008). Some studies have also found that leucine provides amino groups in the amino transduction reaction, which is used to *de novo* synthesis of glutamate (Yudkoff, 2017). In addition, as an activator, Leucine is the most effective amino acid for regulating mTOR (mammalian target of rapamycin) activity. It is well known that mTOR-controlled signaling pathways modulate many integrated physiological functions of the nervous system including neuronal development, synaptic plasticity, memory storage and cognition, thus any factors implicated in deregulation of the mTOR signaling maybe associated with neurological and psychiatric disorders (Jewell et al., 2013; Bockaert and Marin, 2015; Xiao et al., 2015). Moreover, Leucine is the most typical amino acid involved in autophagy regulation, it could activate downstream ULK-1 (Ubiquitin-like proteins-1) and Beclin-1, and then facilitate autophagosome formation following their interaction with LC3 (microtubule-associated protein light chain 3) (Russell et al., 2013). Several studies have demonstrated that Leucine enters the brain from the blood more rapidly than any other as a nutritionally essential amino acid (Yudkoff et al., 2005; Duan et al., 2016), and it competes with kynurenine for blood-to-brain transport and prevents lipopolysaccharide-induced depression-like behavior in mice (Walker et al., 2019), and even maternal immune activation induces dysregulated placental transport of Leucine affecting fetal brain development in rats (Kowash et al., 2022). Meanwhile, our previous result also found that there is an alteration of leucine content in both serum and feces of MS-induced irritable bowel syndrome rats. Disturbance of leucine leads to the development of metabolic diseases associated with neuropathological symptoms, involving the initiation of multiple pathogenic mechanisms and abnormal biochemical reactions. However, there is no existing report on the changes of leucine metabolism in the CSF of rats with MS stress.

Autophagy, a process of cellular self-digestion, delivers intracellular components including superfluous and dysfunctional proteins and organelles to the lysosome for degradation and recycling, and plays important roles in physiological and pathological conditions (Mizushima et al., 2008). Under normal conditions, autophagy is maintained at a low level and is upregulated after various stimuli (nutritional starvation, hypoxia, hormones, and drugs) (Rubinsztein, 2006). It has been reported that MS stress may further affect cognition and behavior in adulthood by modulating autophagy levels in the hippocampus and prefrontal cortex (Liu C. et al., 2018). Autophagy is considered to be a highly selective cellular clearance pathway associated with the maintenance of cellular tissue homeostasis (Tavernarakis, 2020). Excessive autophagy may also lead to disruption of cellular function (Cao et al., 2021). Maintenance of functional autophagy is essential for cellular and organismal health, and dysregulation of autophagy in any direction, whether too little or too much, can lead to cellular defects and organismal functional decline (Aman et al., 2021).

The purpose of this study was to explore the mechanisms of leucine diet modulation in psychiatric disorders in adult rats caused by MS. In summary, we explored the relationships between CSF leucine and cognitive behavior and hippocampal neuron number in rats, and investigated the potential mechanism of early life stress-induced psychiatric disorders through the regulation of neuronal autophagy by leucine.

## Experimental procedures

### Experimental animals

The Sprague Dawley (SD) rat pups were purchased from SiPeiFu Biotechnology Co. and housed with their dams in plastic cages and provided with food and water *ad libitum*. Male neonatal rats of similar body weight in each litter were selected for the experiment. Each litter of neonatal rats was randomly divided into a control group (CON) and a maternal separation group (MS). All of the experiments were approved by the Institutional Animal Care and Use Committee of the Academy of Military Sciences (Permit No: IACUC-DWZX-2020-670).

### Maternal separation

Newborn rats were born with a birth diary of PND1, and for the next 20 days, dams were separated from their littermates for 3 h per day, and the rats were kept at a living environment temperature of 28 ± 2°C. The control group was not separated and was kept in the same environment as the separated group. All of the rats were fed until the weaning period of PND22, and only male rats were used in the present study to avoid variations due to hormonal cycles at PND30. After normal feeding until adult PND60, experiments were conducted and body weight and feed consumption were recorded regularly.

### Dietary leucine formulation

The semi purified diets used in this study were equivalent to diets produced by the American Institute of Nutrition (AIN-93G) (Reeves et al., 1993). All diets were isocaloric and compositionally the same in terms of carbohydrate and lipid component, and were obtained from SiPeiFu Biotechnology Co. The control diet (N) contained 18% protein, 63% carbohydrate, 7% fat, 5% fibers, and 2% L-leucine. The leucine-rich diet (H-LEU) and leucine-deficient diet (L-LEU) contained 5% and 0.22% L-leucine, respectively (Xiao et al., 2011; Viana and Gomes-Marcondes, 2013; Liu T. et al., 2018), and same amount carbohydrate, fat and fibers percentage as the control diet.

### Open field test

Rats were housed in open boxes of size 100 × 100 cm, with the inner walls and bottom painted black and the bottom divided into 25 20 × 20 cm squares. To eliminate the influence of the external environment on the rats, the experiments were conducted in a dedicated laboratory shielded from external sound, and the room temperature was maintained at 22 ± 2°C. To reduce the effect of rat odor, the open-air box was cleaned promptly after the previous rat finished the experiment, and then the next rat experiment was conducted. At the beginning of the experiment, the rat was placed in the center of the open field box, and the open field score, which was the sum of the number of squares crossed by the rat’s nose (crossing) and the number of hind limb stands (rearing), was recorded within 5 min. The rats’ autonomy and exploration behaviors in unfamiliar environment were evaluated comprehensively.

### Morris water maze test

The Morris water maze test was performed in a pool with a diameter of 1.2 m and a water temperature of 25°C. Before the formal test, the subject rats were required to complete five training sessions of finding the platform for 3 min each, once every 12 h. During the training, the water level was higher than the platform to ensure that the rat’s body was mostly on the water surface after successfully finding the platform. During the training phase, the rat was randomly placed in one of the four quadrants of the water maze with its head facing the inner wall. The time taken by the rat to find the platform hidden under the water surface was recorded as the latency to find the platform (escape latency). If the 3-min internal platform search failed, the rat was led to the platform. During the test phase, the underwater platform was removed, and the time to swim to the original underwater platform location after entering the water and the time to traverse the platform area within 3 min (platform passes) were recorded. This experiment was used to evaluate the spatial learning and memory ability of the subject rats.

### Novel object recognition test

The object identification box was a 60 × 60 cm box with the interior and bottom painted black. The rats were acclimatized in the open box for 10 min the day before, and on the second day, two clean blocks of the same color and shape were placed 20 cm apart in the open box to record the cumulative objects explored by the rats in 10 min. One hour later, a clean block of completely different color and shape was replaced in the box, and the exploration time of the rat exposed to the new object (T_*new*_) and the old object (T_*old*_) within 10 min was recorded. The cognitive index = (T_*new*_–T_*old*_)/(T_*new*_ + T_*old*_). This experiment reflected the ability of rats to recognize and remember new things. To avoid odor interference from previous rats, each rat was thoroughly cleaned and disinfected in an open box at the end of the experiment.

### Measurement of leucine in cerebrospinal fluid

After completion of the behavioral test, anesthesia was administered with a combination of urea and chloral hydrate at a rate of 5 ml/kg according to body weight. In prone position, the posterior cervical muscles were blunted and separated to expose the foramen magnum of the occipital bone. The needle was inserted vertically, and a 200 μL microcentrifuge tube was inserted to allow cerebrospinal fluid to flow. The fluid was stored at 80°C. We placed 10 μL of cerebrospinal fluid sample in a microcentrifuge tube, added 40 μL of isotopic internal standard methanol solution, and shook it at 1200 rpm for 5 min. We then aspirated 10 μL of supernatant into a 1.5 mL EP tube and added 70 μL of borate buffer and 20 μL of LAQC derivatization reagent. The supernatant was shaken for 10 min at 55°C and 1200 rpm in a metal bath. The amino acids in the cerebrospinal fluid were detected by ultra-performance liquid chromatography–tandem mass spectrometry (Xevo TQ-S, Waters Corp., Milford, MA, USA).

### Nissl staining

Rats were dissected on ice, heads were cut off, dura mater was separated, skulls were stripped, and brains were isolated. Tissues were preserved in 4% paraformaldehyde, dehydrated, and sealed with wax, and tissues were embedded. The tissues were chilled on a 20° freezing table, and the solidified wax blocks were removed and trimmed; tissues were cut into sections 4 μm thick, affixed to glass slides, and baked in a 60°C oven. The sections were immersed in toluidine blue (Solarbio) for 5 min, then washed with distilled water, slightly fractionated with 1% glacial acetic acid, made transparent in xylene for 5 min, and sealed with neutral glue. Microscopic observation and photography were performed using ImageJ (version 1.45) image analysis software.

### Transmission electron microscopy

The rat brain was placed on an ice plate, and the left and right cerebral cortex were bluntly separated along the median sagittal line to expose the bilateral hippocampus. The maximum volume of tissue was 1 mm × 1 mm × 1 mm. The tissue was fixed with 0.1 m phosphate buffer PB (PH7. 4) 3 times for 15 min at 4°C, fixed at room temperature for 2 h, dehydrated, infiltrated with working solution (acetone:812 embedding agent = 1:1) for 2 h and another working solution (acetone:812 embedding agent = 2:1) for 5 h, transferred to an embedding plate for 5 h, and baked at 37°C for 12 h. The sections were baked and polymerized at 60°C for 48 h. The sections were double stained with uranium and lead and air dried at room temperature for 12 h. Images were acquired using a transmission electron microscopy.

### Western blot analysis

A fully automated protein expression analysis system (WES) was used for the assay. Rat hippocampus tissues were taken, homogenized with an electric homogenizer, lysed by adding cell lysis solution, and centrifuged at 12,000 × *g* for 5 min at 4°C in a low-temperature, high-speed centrifuge; then, the supernatant was removed and set aside, and the samples were quantified with the BCA protein quantification kit. Based on the quantification results, the samples were adjusted to the recommended sample concentration of 2 μg/μL using the WES kit 0.1 × sample buffer. Next, 3 μL of prepared samples, 1 μL of Mastermix, 10 μL of primary antibodies Beclin-1, Lc3, Bcl-2, Bax, and β-actin (1:100, ABclonal), horseradish peroxide 10 μL (HRP), rabbit secondary antibody 10 μL, and luminescent solution 15 μL were loaded onto WES plates. After centrifugation (2500 g, 5 min), the WES plates (12–230 kDa, 25 capillaries) were loaded into a fully automated protein expression analysis system, and the results were analyzed using Compass for WES software (version 4.10). The grayscale values of the bands given by the software were used to calculate the relative protein expression, using β-actin as an internal reference.

### Immunofluorescence

HT-22 cells were inoculated into 24-well plates, ensuring a cell concentration of 1 × 10^5^/ml and 5% CO2, and cultured for 24 h at 37°C using custom leucine-deficient DMEM high-glucose medium. Infection with a fluorescent double-labeled lentivirus mRFP-GFP-LC3 system, MOI = 100, was observed under a fluorescent microscope.

### Lactate dehydrogenase release detection

We added 50 μL of cell suspension to a 96-well plate and incubated it overnight in 5% CO2 in a 37°C cell incubator to allow cells to grow against the wall. We added 10 μL of lysate to high-control wells and incubated them in 5% CO2 in a 37°C cell incubator for 30 min. After adding 100 mL of working solution per well, we incubated the samples at room temperature and protected them from light. After adding 50 μL of termination solution per well, we measured the absorbance at 490 nm with an enzyme marker within 5 min.

### Flow cytometry

HT-22 cells were digested and inoculated into 6-well plates at a density of 2 × 10^5^ cells/mL, 3 mL per well. When cells reached 80–90%, they were given serum-free treatment and various factor interventions, such as leucine and B-A1. The medium in the 6-well plates was added to round-bottom flow tubes and labeled; cells in the wells were washed with PBS and digested with 500 μL of 0.25% EDTA-free trypsin at room temperature. We digested the cells for 3 min and added 500 μL medium to the flow tube to terminate the digestion, blew gently, pipetted into the flow tube, centrifuged at room temperature for 5 min using a medical centrifuge at 1000 r/min, discarded the supernatant, added 1 mL PBS for vortex shaking, centrifuged twice at room temperature for 5 min using a medical centrifuge at 1000 r/min, stained the samples according to the instructions of the Annex V and PI double-staining kit, and tested them in the machine after 30 min of protection from light.

### Statistical analysis

Statistical software SPSS 26.0 was used for statistical analysis, and quantitative results were expressed as mean ± standard deviation (sd). Quantitative values between two groups were compared using the independent samples *t*-test at the level of α = 0.05. One-way ANOVA was used for multiple groups, and *p* < 0.05 was considered a statistically significant difference. Nissl staining was processed and analyzed with ImageJ software.

## Results

### Maternal separation caused cognitive dysfunction in sprague dawley rats

**FIGURE 1:**
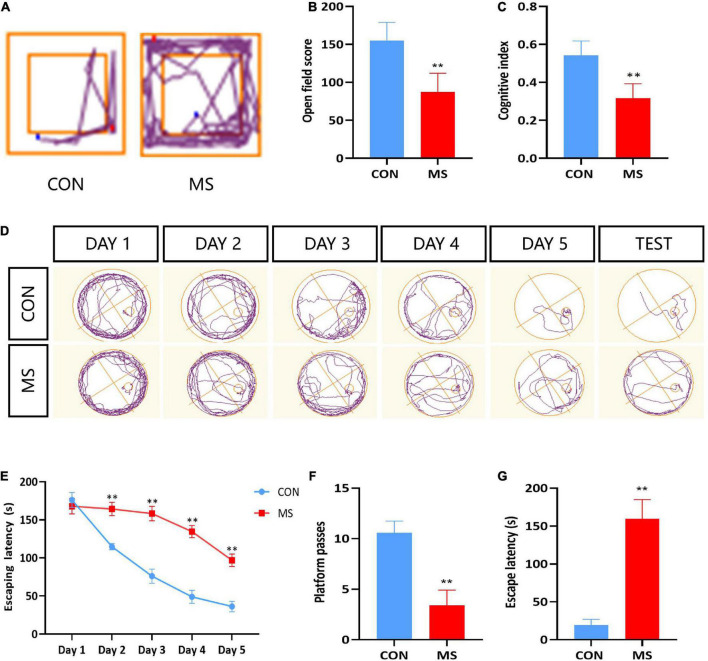
MS effects on recognition dysfunction in behavior test. **(A)** Trajectory of open field test. **(B)** Open field test. **(C)** Novel object recognition test. **(D)** Trajectory of Morris water maze. **(E)** Escape latency of Morris water maze during learning time. **(F)** Platform passes of Morris water maze. **(G)** Escape latency of Morris water maze. ^**^p < 0.01 vs. control, *n* = 5.

Behavioral tests were conducted to investigate the effects of MS stress on cognitive function. Compared with the CON group, the results showed significantly lower cognitive function in the MS group, including self-exploratory ability, novel object recognition, and spatial learning memory in the open field, novel object recognition test, and Morris water maze test (*p* < 0.05, *p* < 0.01; Figures 1A–G).

### Maternal separation decreases the number of hippocampal neurons, which is positively correlated with leucine

**FIGURE 2:**
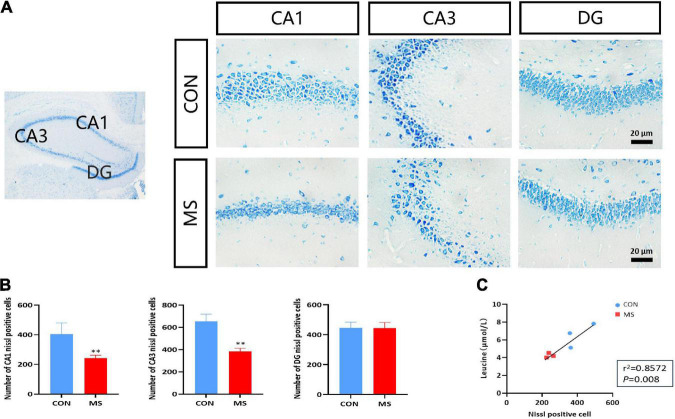
Hippocampal neuronal damage is caused by MS, and the number of neurons is positively correlated with leucine content in cerebrospinal fluid. **(A)** Nissl staining in the hippocampus. **(B)** Statistics of Nissl-stained positive cells. **(C)** Leucine levels were highly correlated with the number of hippocampal neurons. ^**^p < 0.01 vs. control, *n* = 3.

To confirm whether there was neuronal damage in the hippocampal region associated with MS-induced cognitive impairment in rats, we used Nissl staining to stain neuronal cells (mainly neurons) in the hippocampal region, and the results showed that neurons in the hippocampal CA1 and CA3 regions of MS rats were loosely arranged, lightly stained, and reduced in number compared with the CON group, with no differential changes in the DG region (*p* < 0.05, *p* < 0.01; Figures 2A,B). The leucine levels in CSF were significantly decreased in MS rats, and there was a positive correlation with the number of hippocampal neurons (*p* < 0.05, *p* < 0.01; Figure 2C).

### Leucine supplementation mitigates cognitive dysfunction caused by maternal separation

**FIGURE 3:**
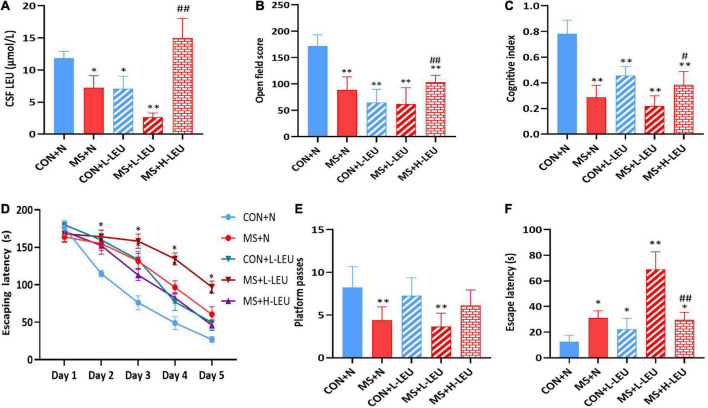
Effect of dietary leucine content on cognitive function in rats. **(A)** Leucine content in cerebrospinal fluid. **(B)** Open field score. **(C)** Cognitive index. **(D)** Escape latency of Morris water maze during learning time. **(E)** Platform passes of Morris water maze. **(F)** Escape latency of Morris water maze. *p < 0.05, ^**^p < 0.01 vs. CON + N, ^#^*p* < 0.05, ^##^*p* < 0.01 vs. MS + L-LEU vs. control, *n* = 5.

To study the effect of different levels of leucine on cognitive function in MS animal models, after MS, the rats were fed a normal leucine diet (N, 2%), low leucine diet (L-LEU, 0.22%), or high leucine diet (H-LEU, 5%) for 40 days. A stress model of MS rats was established, and rats were grouped into CON + N (control normal diet), CON + L-LEU (control low-leucine diet), MS + N (stress group normal diet), MS + L-LEU (stress group low-leucine diet), and MS + H-LEU (stress high-leucine diet) groups. At PND 60, leucine in CSF was measured, and cognition-related behavioral tests were performed. The results showed that, compared to the control normal diet group, the leucine levels were lower in the low leucine diet, MS normal diet, and MS low-leucine diet rats, while the MS high-leucine diet group had higher leucine levels than the MS low-leucine diet group (*p* < 0.05, *p* < 0.01; Figure 3A). Rats in the low-leucine diet group had lower scores in both the open field test and the novel object recognition test compared to the control normal diet group, and the MS high-leucine diet group had higher scores than the MS low-leucine diet group (*p* < 0.01; Figures 3B,C). The MS normal diet group and MS low-leucine diet group had higher scores during the assessment phase (*p* < 0.05, *p* < 0.01; Figure 3D). The number of stage penetrations was reduced in both the MS normal diet group and the MS low-leucine diet group compared with the control normal diet group, and there was a tendency toward an increased number of stage penetrations in the MS high-leucine diet group. The low-leucine diet caused a prolonged latency time in all groups of rats compared with the control normal diet group, and there was a decreased latency time in the MS high-leucine diet group compared with the MS low-leucine diet group in male rats (*p* < 0.05, *p* < 0.01; Figures 3E,F).

### Leucine-deficient diet and maternal separation stress activate hippocampal neuronal autophagy

**FIGURE 4:**
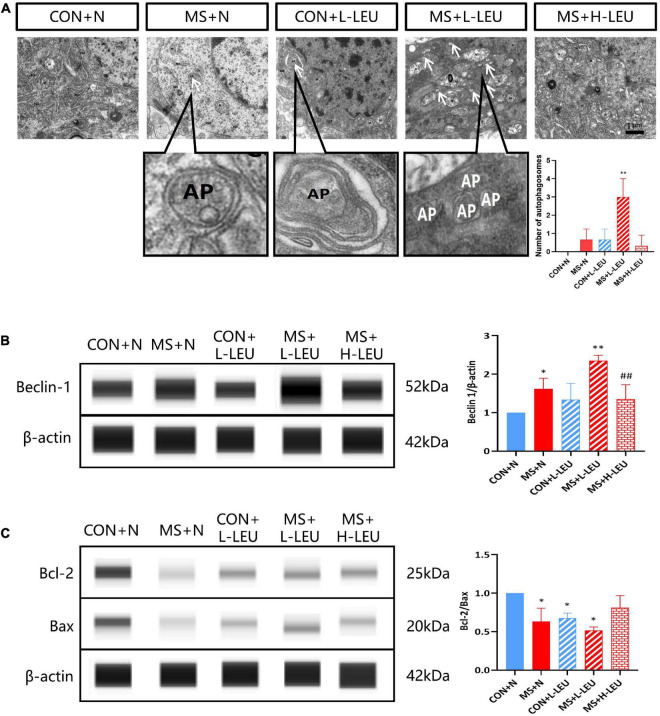
Leucine plays an important role in the development of cognitive impairment induced by maternal separation stress. **(A)** The autophagosomes in the hippocampus were detected by a transmission electron microscopy. Existence of the cytoplasmic components enclosed in double-membraned vesicles is the hallmark of autophagosomes (Eskelinen, 2008). **(B)** Beclin-1 in the hippocampus was detected by WES. **(C)** Bcl-2 and Bax were detected by WES. *p < 0.05, ^**^p < 0.01 vs. CON + N, ^##^*p* < 0.01 vs. ME + L-LEU, *n* = 3.

To investigate the effect of MS stress on rat hippocampal autophagy, samples were taken and observed using electron microscopy, which showed that autophagosomes were present in the hippocampus of the low-leucine diet CON and MS groups, and no autophagic vesicles were found in the high-leucine diet MS group, as shown in Figure 4A. The hippocampus of rats was taken to detect Beclin-1 expression, which was increased in the normal diet and low-leucine diet MS group but decreased in the high-leucine diet MS group compared with the low-leucine diet MS group (*p* < 0.05, *p* < 0.01; Figure 4B). The Bcl-2/Bax ratio was decreased in the low-leucine diet CON group, normal diet MS group, and low-leucine diet MS group (*p* < 0.05; Figure 4C).

### Leucine deficiency induces elevated autophagy with damaging effects on neurons

**FIGURE 5:**
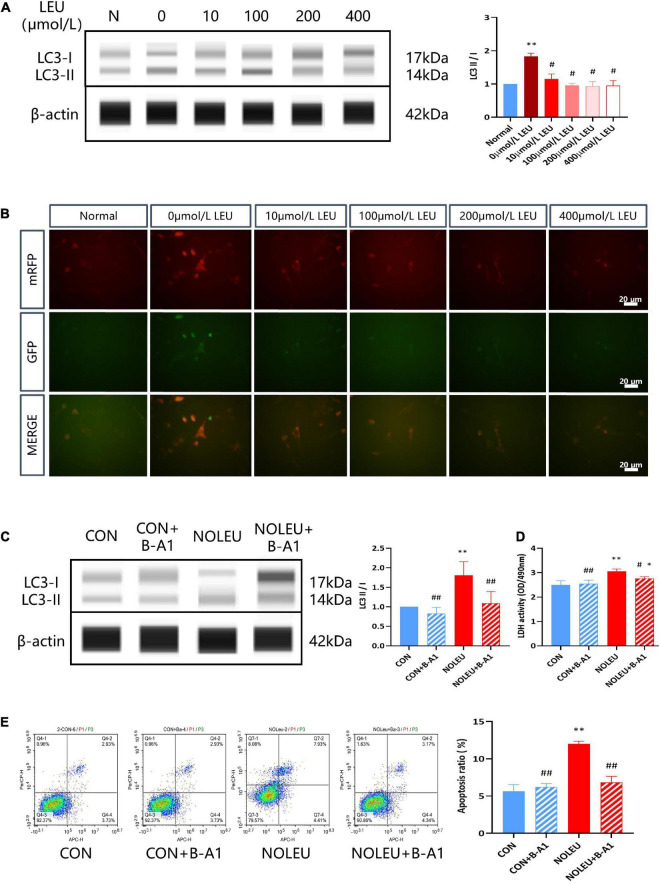
Leucine deficiency can activate neuronal autophagy. **(A)** LC3II and LC3I were detected by WES *in vivo*, ^**^p < 0.01 vs. Normal, *^#^p* < 0.05 vs. 0 μmol/L LEU, *n* = 3. **(B)** mRFP-GFP-LC3 was detected by IF. **(C)** Effects of autophagy inhibitors and leucine deficiency on the expression of LC3II and LC3I in leucine-deficient cultured neurons. **(D)** Effects of autophagy inhibitors and leucine deficiency on LDH release from leucine-deficient cultured neurons. **(E)** Effects of autophagy inhibitors and leucine deficiency on neuronal apoptosis. *p < 0.05, ^**^p < 0.01 vs. CON, ^#^*p* < 0.05, ^##^*p* < 0.01 vs. NOLEU, *n* = 3.

To further verify the role of neuronal autophagy upregulation by leucine deficiency in ELS-induced metal disorder rats, HT-22 stress cell models were cultured in a specially customized cell culture medium with different contents of leucine and autophagy inhibitor. The results showed increased expression of LC3II/I in leucine starvation neurons compared to the normal (routine culture of neural cells) group, and it was remarkably restored after adding different concentrations of leucine (*p* < 0.05; Figure 5A). Autophagy double-labeled lentivirus (mRFP-GFP-LC3) infection of neurons cultured with different concentrations of leucine medium showed enhanced bicolor fluorescence in the Leucine starvation group, which diminished with increasing leucine concentration, as shown in Figure 5B. Furthermore, leucine-deficient culture and autophagy inhibitor intervention in neurons were examined for LC3 protein expression, lactate dehydrogenase (LDH) release, and cell apoptosis. The results showed that the LC3II/I ratio, LDH activity, and cell apoptosis rate in the NOLEU group were markedly elevated compared to the CON group, but they were significantly decreased after treatment with the autophagy inhibitor B-A1 (*p* < 0.05, *p* < 0.01; Figures 5C–E).

## Discussion

Separation from the mother early in the animal’s life is a traumatic event and is a major cause of various psychiatric disorders in adulthood, predisposing individuals to cognitive impairment and abnormal emotional states (Felitti et al., 1998). In rodent MS studies, it was found that MS stress lasting 3–6 h per day for 14-21 days after birth, as a classical form of early-life adverse stress modeling, can cause abnormalities in a variety of cognitive functions in adulthood, including the ability to remember new and unfamiliar spatial learning, fear memory, and emotion (Aisa et al., 2007). We used the open field test, object recognition test, and Morris water maze test to evaluate the cognitive function of MS stress rats and found that MS rats’ spatial autonomic exploration ability, object recognition memory ability and spatial learning memory ability were all decreased, which was similar to previous results (Yang et al., 2019). Cognitive impairment in rats is largely associated with damage to hippocampal neurons and its reduced density in the brain, and a work reported that Nissl staining can stain most neurons and a few glial cells as well as found a significant loss of neurons in the dentate gyrus of the hippocampus in MS mice (Fabricius et al., 2008). Our results also revealed a reduced number of neurons in CA1 and CA3 regions of the hippocampus of MS-stressed rats with a disorganized and sparse arrangement.

Previous studies have found that MS can cause significant changes in a variety of amino acids in the peripheral and central parts of the body (Zhao and Qian, 2014; Liang et al., 2018; Xiao-tian et al., 2022) and is closely associated with the occurrence of a variety of stress-related biological damage effects (Griffin and Bradshaw, 2017). Recent works have identified sex difference in response to early life stress. However, some reported that early life stress models in rodents cannot replicate the effects of gender factors, that may be due to female rodents are susceptible to differences in estrus cycles (Bath et al., 2017; Goodwill et al., 2019; Le et al., 2022). Our previous data also found that there are sex selective effects on cognitive function, hippocampal structure and amino acids metabolism in MS rats, suggesting that threonine in female rat CSF maybe play a critical role in cognitive and emotional impairment caused by MS. Thus, only male rats were used in the present study to avoid variations due to hormonal cycles. We measured leucine content in rat CSF and found there was a positive correlation with the number of hippocampal neurons. Meanwhile, detection of anti-apoptosis-related protein Bcl-2 and pro-apoptosis-related protein Bax expression revealed that a low-leucine diet and MS stress induced an increased in neuron apoptosis in the hippocampus. Modulation of cognitive function in animals through adjustment of nutrient ratios in the diet is a classical treatment for neuropsychiatric disorders and has been proven effective in a variety of neurodegenerative diseases (Meeusen and Decroix, 2018; Onaolapo et al., 2019). Leucine can rapidly enter the brain from the periphery through the blood-brain barrier (Murín and Hamprecht, 2008). In our study, we found that insufficient leucine levels exacerbated cognitive impairment in MS rats in adulthood, while cognitive function could be recovered to some extent by providing a high leucine diet, suggesting that the modulation of cognitive function by leucine in MS rats may be affected by multiple factors.

Autophagy is a highly conserved eukaryotic cellular recycling process and is important to maintain cellular homeostasis. That is either hyper- or hypo-autophagy can be deleterious, a complexity seen in its dual role in cytoprotection and cell death. Growing evidence reveals that alterations in autophagy occur in many human diseases, including neuronal death and survival (Kim and Lee, 2014; Mizushima and Levine, 2020). Abnormal leucine metabolism in MS rats may affect cognitive function by regulating neuronal autophagy and then resulting in a decrease in the number of neurons. Electron microscopic results showed that both low leucine and MS stress could activate hippocampal autophagy, which may be related to leucine reduction in rat CSF caused by MS stress. Meanwhile, there is a significant increase in the expression of autophagy-related protein Beclin-1, which was basically consistent with the electron microscopic results. Leucine is a critical mTORC1 (rapamycin complex 1) regulator in response to nutritional status and a variety of stress signals, and the latter is a well-conserved negative regulator of autophagy. Leucine deficiency regulates autophagosome biogenesis via its metabolite acetyl-coenzyme A (AcCoA) and stimulates autophagy in cells via mTORC1 inhibition (Son et al., 2020). When mTOR is inhibited by leucine-deficient signaling, the upregulation of the LC3II/I ratio in LC3 (microtubule-associated light chain protein 3), a widely present marker of autophagosomes and autophagolysosomes, may have the potential for both autophagy activation and inhibition of the downstream pathway of autophagy (Kuma et al., 2007), thus it important to precisely detect autophagic flow and to investigate which function it performs in neurons (Kimura et al., 2008). Using the autophagy double-labeled lentiviral mRFP-GFP-LC3 system to track autophagy morphologically (Mizushima et al., 2010), we showed that leucine-deficient cultured neurons with a significantly elevated LC3II/I ratio and enhanced autophagy double-labeled mRFP-GFP-LC3 lentiviral two-color fluorescence, indicating that leucine deficiency can induce autophagic activation in neurons.

In recent decades, autophagy has been found to help fight against a variety of human diseases, but, at the same time, autophagy can also promote the procession of certain pathologies. Excessive autophagy not only fail to protect cells, but also leads to cell death, degrades the nucleus, activates apoptosis, and eventually causes damage (Zeng et al., 2020). Autophagy is also known as type II programmed cell death and is interactively linked to apoptosis (Ghavami et al., 2014). In this study, Bcl-2 was downregulated and Beclin-1 was upregulated, which may be related to the activation of autophagy by leucine deficiency and inhibition of Bcl-2, thus activating apoptosis. Although most studies showed that autophagy has a protective effect on neurons, but the existence of autophagic cell death (ACD) indicated that cell death is also executed through autophagy, such as an increase in autophagic flow and autophagic markers is found in dying cells, and inhibition of autophagy by pharmacological inhibitors or genetic methods can also rescue and prevent cell death (Shen et al., 2011; Hensley and Harris-White, 2015). In a variety of disease models, the damage to target organs by pathogenic factors has been further mitigated by pharmacological attenuation of autophagy levels (Li et al., 2017; Xu et al., 2022). LDH and apoptosis assays have been used in several studies to measure the extent of cell damage (Kumar et al., 2018; Zhang et al., 2020), as previously described, neurons cultured in leucine-deficient medium showed a significant increase in cellular LDH release and apoptosis, and after administration of autophagy inhibitors, cellular LDH release and apoptosis decreased significantly, suggesting that leucine deficiency induces excessive autophagy in neurons and then causes damage. It does seem likely that the coordinated regulation of autophagy and apoptosis may underlie diverse aspects of tissue homeostasis and disease pathogenesis.

In conclusion, MS leads to the decrease of leucine content in the rat CSF, which may mediate the excessive activation of autophagy in hippocampal neurons to induce neuron damage and apoptosis and then result in cognitive impairment. Abnormal autophagy may be an important pathological mechanism of cognitive dysfunction induced by ELS, and leucine may be regarded as a potential pharmacological target for improving the mental health status.

## Data availability statement

The raw data supporting the conclusions of this article will be made available by the authors, without undue reservation.

## Ethics statement

The animal study was reviewed and approved by Animal Care and Use Committee of the Academy of Military Sciences.

## Author contributions

XTW conducted the research, performed the analysis, and wrote the manuscript. XW and FX analyzed the data and reviewed the manuscript. ZS, BG, FL, SW, YW, and YT performed the experiments. LQ and YZ designed the research, supervised the research, reviewed and revised the manuscript, and granted the research funds. All authors contributed to the article and approved the submitted version.
